# Robustness of Wave–Particle Duality under Unruh Effect

**DOI:** 10.3390/e26010001

**Published:** 2023-12-19

**Authors:** Pedro H. M. Barros, Irismar G. da Paz, Olimpio P. de Sá Neto, Helder A. S. Costa

**Affiliations:** 1 Departamento de Física, Universidade Federal do Piauí, Teresina 64049-550, PI, Brazil; phmbarros@ufpi.edu.br (P.H.M.B.); irismarpaz@ufpi.edu.br (I.G.d.P.); 2 Coordenação de Ciência da Computação, Universidade Estadual do Piauí, Parnaíba 64202-220, PI, Brazil; olimpioqedc@gmail.com

**Keywords:** Unruh effect, coherence, complementarity, wave–particle duality

## Abstract

By considering a uniformly accelerated two-level system in an initial superposition state of a qubit, we investigate the loss of coherence induced by the acceleration. In addition, we investigate the impact of acceleration on the complementarity relation in a quantum interferometric circuit or quantum scattering circuit. We present an alternative approach to exploring acceleration effects through examination of quantum coherence decay and degradation in the interference pattern. Our investigations help to provide understanding of the consequences of decoherence induced by the Unruh effect on the wave–particle duality of a uniformly accelerated qubit.

## 1. Introduction

Quantum coherence is a fundamental concept in quantum physics and a key resource for a wide range of quantum technologies. It enables the exploitation of quantum effects such as superposition and interference, which are the basis for the advantages of quantum computing [1,2], quantum communication [3], quantum metrology [4,5,6], and quantum information processing [7], among others. However, coherence is a fragile property. It can be easily disrupted by interactions with the environment: a process known as decoherence. Environmental factors, such as the Unruh effect [8,9,10,11], can introduce random fluctuations and disturbances, leading to a loss of coherence in the quantum system. For example, in ref. [12], the authors studied quantum coherence behavior in a non-inertial frame. In particular, they found that the acceleration of the observers can be treated as a kind of “environmental decoherence”, and the effect degrades the quantum correlation from the perspective of the accelerated observers. In addition, numerous ideas have studied the dynamics of quantum coherence for an accelerated atom. They include topics such as quantum coherence for an accelerated atom immersed in an electromagnetic field [13,14], entanglement harvesting [15,16,17], relativistic quantum computing [18,19], quantum communication [20,21], relativistic quantum optics [22], etc. This research has both fundamental and practical applications.

Exploring the degradation of quantum features under acceleration is essential for elucidating the fundamental limits and potential mitigations in relativistic quantum systems and offers insights into the design and implementation of future relativistic quantum technologies. As noted by Benatti and Floreanini [23], quantum fluctuations of the background field act as an environment. The Unruh thermal bath, according to the authors, causes decoherence and destroys the properties of coupled Unruh–DeWitt detectors. With the Unruh effect being comparable to a decoherence channel, this work aims to investigate the impact of decoherence induced by the Unruh effect on the wave–particle duality of a uniformly accelerated qubit. Therefore, we consider the uniformly accelerated qubit as a probe system that interacts with the vacuum and field fluctuations as its “environment”. In this scenario, if we solely observe the detector’s state rather than the field, we will observe it in either the excited or ground state. Consequently, the detector’s state ceases to be pure and instead becomes a statistical mixture due to interaction with the scalar field, signifying a loss of coherence. Our results imply that the Unruh effect inevitably disturbs the internal state of the accelerated qubit and causes some loss of wave–particle duality information.

The paper follows the following structure: First, we introduce the standard Unruh–DeWitt detector model, which describes the coupling between the detector (a two-level system) and the quantum scalar field. Additionally, we assess finite-time corrections to the transition probability rate. Second, we outline our proposed approach, which involves a uniformly accelerated detector prepared in a superposition state that interacts with a massless scalar field, followed by measurement of its internal states. We quantify the extent of the loss of coherence using the $l^{1}$ norm coherence, which measures the sum of absolute values of the off-diagonal elements of the detector’s density matrices. Subsequently, we examine the effects of acceleration on the wave–particle duality of a uniformly accelerated qubit in a quantum interferometric circuit. Finally, the last section is devoted to a brief conclusion. Throughout this paper, natural units ($\hbar =c=k_{B}=1$) are used.

## 2. Fulling–Davies–Unruh Effect

In the mid-1970s, Unruh and Wald [8] proposed a scenario involving a uniformly accelerated detector, such as a two-level atom, coupled to a massless scalar field in a Minkowski vacuum. They discovered that the detector perceives a thermal distribution of particles, with the temperature of this distribution being directly proportional to the detector’s proper acceleration. This phenomenon, widely known as the Fulling–Davies–Unruh effect or simply the Unruh effect [9,10,11], has since been extensively studied. The original analysis of the Unruh effect employed an in–out approach, assuming that the initial state of the system, composed of the detector and the quantum scalar field, was prepared at the infinite past, while the final state was evaluated at the infinite future. However, subsequent investigations [24,25,26] have explored the response of uniformly accelerated detectors when coupled to the quantum scalar field for a finite duration. To assess the response of the Unruh–Dewitt detector as a function of the interaction time, we consider a detector with internal levels |g〉 and |e〉, which is coupled to a real, free, and massless scalar field ϕ through the interaction Hamiltonian $H_{int}$ in the detector’s frame:

(1) $$\begin{array}{c} H_{int}=g\Theta \left(\tau \right)\mu \left(\tau \right)\phi \left[x\left(\tau \right)\right], \end{array}$$

where τ is the detector’s proper time, μ(τ) is known as the monople moment of the detector, and *g* is a small coupling constant. In order to describe finite-time interactions, we assume that Θ(τ) is a gradual window function with the properties: Θ(τ)≈1 for |τ|≪T and Θ(τ)≈0 for |τ|≫T, where *T* is the interaction time. Let us assume that the detector follows a trajectory x(τ) while the field is in the Minkowski vacuum state $|0_{M}\rangle$. Up to the first-order perturbation theory, the probabilities that the detector absorbs or emits a quantum of energy are given, respectively, by

(2) $$\begin{array}{c} P^{-}=g^{2}{\left|\langle g|\mu \left(0\right)|e\rangle \right|}^{2}F^{-}, \end{array}$$

(3) $$\begin{array}{c} P^{+}=g^{2}{\left|\langle e|\mu \left(0\right)|g\rangle \right|}^{2}F^{+}, \end{array}$$

where the response function $F^{\pm }$ encodes information about the motion of the detector, the initial state of the field, and the on/off switch of the detector. $F^{\pm }$ can be expressed as:

(4) $$\begin{array}{c} F^{\pm }=\int_{-\infty }^{\infty }d\tau \int_{-\infty }^{\infty }d\tau^{\prime }\Theta \left(\tau \right)\Theta \left(\tau^{\prime }\right)e^{\pm i\Omega (\tau -\tau^{\prime })}G^{+}\left[x\left(\tau \right),x\left(\tau^{\prime }\right)\right] \end{array}$$

where $G^{+}\left[x\left(\tau \right),x\left(\tau^{\prime }\right)\right]=\langle 0_{M}|\phi \left[x\left(\tau \right)\right]\phi \left[x\left(\tau^{\prime }\right)\right]|0_{M}\rangle$ is the Wightman function [27]. Note that the factors before $F^{-}$ and $F^{+}$ in Equations (2) and (3) depend only on the internal structure of the detector, and we will drop them hereafter and refer to $F^{-}$ and $F^{+}$ as transition probabilities. For inertial and accelerated trajectories in Minkowski space, the Wightman function is invariant under time translation in the detector frame, i.e., $G^{+}\left[x\left(\tau \right),x\left(\tau^{\prime }\right)\right]=G^{+}\left(\tau -\tau^{\prime }\right)$ [28,29]. By using the following property

$$f\left(u\right)\left[e^{-i\Omega u}G^{+}\left(u\right)\right]=f\left(-i\frac{\partial }{\partial \Omega }\right)\left[e^{-i\Omega u}G^{+}\left(u\right)\right],$$

for any function f(u) that has a power series expansion around u=0, we can provide an asymptotic expression for the probability of transition with any analytic window function as

$$F^{\pm }=\Theta \left(i\frac{\partial }{\partial \Omega }\right)\Theta \left(-i\frac{\partial }{\partial \Omega }\right)F^{\pm }\left(\infty \right),$$

where $F^{\pm }\left(\infty \right)$ corresponds to the infinite-time detector

(5) $$\begin{array}{c} F^{\pm }\left(\infty \right)=\int_{-\infty }^{\infty }d\tau \int_{-\infty }^{\infty }d\tau^{\prime }e^{\pm i\Omega (\tau -\tau^{\prime })}G^{+}\left(\tau -\tau^{\prime }\right). \end{array}$$

Expanding Θ(τ) as a Taylor series around τ=0 and assuming that Θ(0)=1 and $\Theta^{\prime }\left(0\right)=0$, we obtain that $F^{\pm }\approx F^{\pm }\left(\infty \right)-\Theta^{\prime \prime }\left(0\right)\frac{\partial^{2}F^{\pm }\left(\infty \right)}{\partial \Omega^{2}}$. The corresponding transition probability per unit time is given by $R^{\pm }\approx R^{\pm }\left(\infty \right)-\Theta^{\prime \prime }\left(0\right)\frac{\partial^{2}R^{\pm }\left(\infty \right)}{\partial \Omega^{2}}$, where $R^{\pm }\left(\infty \right)=\int_{-\infty }^{\infty }d\Delta \tau e^{\pm i\Omega \Delta \tau }G^{+}\left(\Delta \tau \right)$ with $\Delta \tau =\tau -\tau^{\prime }$. It is worth noting that the transition probability depends on the derivatives of the window function. Consequently, if the detector is turned on and off abruptly, these derivatives can lead to divergent responses. To avoid such divergences, we consider the Gaussian window function $\Theta \left(\tau \right)=\exp\left(-\frac{\tau^{2}}{2T^{2}}\right)$, which smoothly switches the detector on and off. In this case, to the leading order, the finite-time corrections to the transition probability rate can be expressed as:

(6) $$\begin{array}{c} R^{\pm }\approx R^{\pm }\left(\infty \right)+\frac{1}{2T^{2}}\frac{\partial^{2}R^{\pm }\left(\infty \right)}{\partial \Omega^{2}}+O\left(\frac{1}{T^{4}}\right). \end{array}$$

Here, $R^{+}$ and $R^{-}$ correspond to the rates of the absorption probability and the excitation probability, respectively. In particular, for a uniformly accelerated detector moving with proper acceleration *a*, the natural coordinate system in the detector frame is related to the Minkowski coordinates by the transformations [30]:

$$\begin{array}{c} t\left(\tau \right)=a^{-1}\sinh\left(a\tau \right),\;z\left(\tau \right)=a^{-1}\cosh\left(a\tau \right),\;x\left(\tau \right)=y\left(\tau \right)=0. \end{array}$$

In terms of these coordinates, the Wightman function can be expressed as $G^{+}\left(\Delta \tau \right)=-\frac{1}{4\pi^{2}}\sum_{n=-\infty }^{\infty }\frac{1}{{\left(\Delta \tau -2i\epsilon +2\pi in/a\right)}^{2}},$ and the rate of transition probabilities $R^{-}\left(\infty \right)$ and $R^{+}\left(\infty \right)$ becomes

(7) $$\begin{array}{ccc} R_{\mathrm{acc}}^{-}\left(\infty \right) & = & \frac{1}{2\pi }\frac{\Omega }{e^{\frac{2\pi \Omega }{a}}-1}, \end{array}$$

(8) $$\begin{array}{ccc} R_{\mathrm{acc}}^{+}\left(\infty \right) & = & \frac{1}{2\pi }\frac{\Omega e^{\frac{2\pi \Omega }{a}}}{e^{\frac{2\pi \Omega }{a}}-1}, \end{array}$$

which is a standard thermal spectrum. This result demonstrates that when a uniformly accelerated detector is placed in the Minkowski vacuum, its response is equivalent to that of a detector at rest in a thermal bath with a temperature proportional to the acceleration. This temperature is known as the Unruh temperature and can be expressed as $T_{U}=\frac{a}{2\pi }$. Substituting (7) into (6), we obtain the following expression:

(9) $$\begin{array}{ccc} R_{\mathrm{acc}}^{-} & \approx & \frac{1}{2\pi }\frac{\Omega }{e^{\frac{2\pi \Omega }{a}}-1}\left\{1+\frac{2\pi }{a\Omega T^{2}}\frac{e^{\frac{2\pi \Omega }{a}}}{{\left(e^{\frac{2\pi \Omega }{a}}-1\right)}^{2}}\left[1-e^{\frac{2\pi \Omega }{a}}+\frac{\pi \Omega }{a}\left(e^{\frac{2\pi \Omega }{a}}+1\right)\right]\right\}, \end{array}$$

(10) $$\begin{array}{ccc} R_{\mathrm{acc}}^{+} & \approx & \frac{1}{2\pi }\frac{\Omega e^{\frac{2\pi \Omega }{a}}}{e^{\frac{2\pi \Omega }{a}}-1}\left\{1+\frac{2\pi }{a\Omega T^{2}}\frac{1}{{\left(e^{\frac{2\pi \Omega }{a}}-1\right)}^{2}}\left[1-e^{\frac{2\pi \Omega }{a}}+\frac{\pi \Omega }{a}\left(e^{\frac{2\pi \Omega }{a}}+1\right)\right]\right\}. \end{array}$$

This is an approximate expression for the rate of transition probabilities when the detector gradually couples to a scalar field over a finite time interval *T*. It is clear from Equations (9) and (10) that in the limit T→∞, the first term of the expression above dominates, and we recover the standard result.

## 3. Setup A: Accelerated Qubit

**Figure 1:**
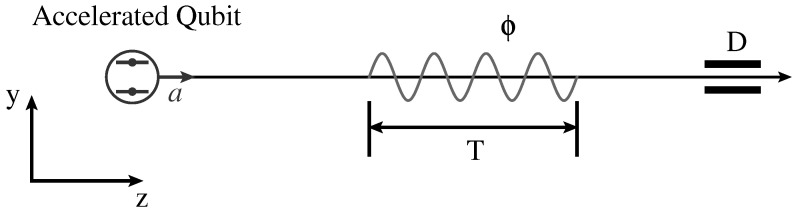
Illustration of a quantum scheme for estimating the loss of coherence of a single qubit induced by the Unruh effect. A qubit, initially prepared in the superposition state $|\psi_{D}\rangle$, undergoes uniform acceleration, interacts with a massless scalar field ϕ for a finite time interval *T*, and subsequently has its internal states measured.

The basic idea of our scheme is summarized as follows. As shown in Figure 1, we consider a setup comprising a two-level system and a free massless scalar field. The field is in the vacuum state $|0_{M}\rangle$, while the two-level system is prepared in the superposition state $|\psi_{D}\rangle =\alpha |g\rangle +\beta |e\rangle$ with ${\left|\alpha \right|}^{2}+{\left|\beta \right|}^{2}=1$, i.e., the two-level system is in a qubit state. It is convenient to rewrite $|\psi_{D}\rangle$ as a Bloch vector:

(11) $$\begin{array}{c} |\psi_{D}\rangle =\cos\frac{\theta }{2}e^{i\chi /2}|g\rangle +\sin\frac{\theta }{2}e^{-i\chi /2}|e\rangle \end{array}$$

where θ∈[0,π] and χ∈[0,2π] are the polar and azimuthal angles in the Bloch sphere, respectively. Following its preparation, the qubit undergoes uniform acceleration in a linear accelerator. It then interacts with the scalar field ϕ for a specific duration of time, denoted as *T*. Finally, the internal states of the qubit are measured.

Let us assume that initially, the system consisting of the accelerated detector and the scalar field is prepared in the state: $|\psi_{\mathrm{in}}\rangle \to |0_{M}\rangle ⊗|\psi_{D}\rangle ,$ where $|0_{M}\rangle$ is the Minkowski vacuum state of the field and is defined in the inertial laboratory frame. Considering that the interaction between the detector and the field is governed by Equation (1) in the weak coupling regime, the unitary transformation induced by the Hamiltonian (1) is given by

(12) $$\begin{array}{c} \hat{U}=I-ig\int_{-\infty }^{\infty }d\tau \Theta \left(\tau \right)\mu \left(\tau \right)\phi \left[x\left(\tau \right)\right]. \end{array}$$

Note that the monople moment of the qubit can be written as $\mu \left(\tau \right)=\left[{\hat{\sigma }}_{+}e^{i\Omega \tau }+{\hat{\sigma }}_{-}e^{-i\Omega \tau }\right]$, where Ω is the transition angular frequency, and ${\hat{\sigma }}_{+}=\left|e\rangle \langle g\right|$ and ${\hat{\sigma }}_{-}=\left|g\rangle \langle e\right|$ are the raising and lowering operators, respectively. Thus, we can deduce that the interaction between the uniformly accelerated detector and the field leads to the following transformation:

(13) $$\begin{array}{cc} |0_{M}\rangle ⊗|g\rangle & \to |0_{M}\rangle ⊗|g\rangle -ig\Phi \left(\tau \right)|0_{M}\rangle ⊗|e\rangle ,\\ |0_{M}\rangle ⊗|e\rangle & \to |0_{M}\rangle ⊗|e\rangle -ig\Phi^{*}\left(\tau \right)|0_{M}\rangle ⊗|g\rangle . \end{array}$$

where $\Phi \left(\tau \right)=\int_{-\infty }^{\infty }d\tau \Theta \left(\tau \right)e^{i\Omega \tau }\phi \left[x\left(\tau \right)\right]$. Note that (13) characterizes the “dressing” of the ground and excited states of the uniformly accelerated detector due to the interaction. In our analysis, we focus solely on observing the effects of acceleration. Therefore, we assume that the lifetime of the excited state is longer than the interaction time. This ensures that the atom does not transition to the ground state by spontaneously emitting a photon into the field [31,32]. Consequently, taking into account Equation (13), the state of the system after the finite-time interaction between the accelerated detector and the field can be expressed as:

(14) $$\begin{array}{ccc} |\psi_{\mathrm{out}}\rangle & \to & |0_{M}\rangle ⊗|\psi_{D}\rangle -ige^{i\chi /2}\cos\frac{\theta }{2}\Phi \left(\tau \right)|0_{M}\rangle ⊗|e\rangle \\ & - & ige^{-i\chi /2}\sin\frac{\theta }{2}\Phi^{*}\left(\tau \right)|0_{M}\rangle ⊗|g\rangle . \end{array}$$

When only the state of the detector is observed, disregarding the field, the detector will be found either in the excited state or the ground state. However, it will no longer be in a pure state. The resulting state can be described by the reduced density matrix ${\hat{\rho }}_{D}={\mathrm{Tr}}_{M}[|\psi_{\mathrm{out}}\rangle \langle \psi_{\mathrm{out}}|]$. In the basis {|g〉,|e〉}, the reduced density matrix of the detector is given by

(15) $$\begin{array}{cc} {\hat{\rho }}_{D} & =N^{-1}[\left(\cos^{2}\frac{\theta }{2}+g^{2}TR_{\mathrm{acc}}^{-}\sin^{2}\frac{\theta }{2}\right)\left|g\rangle \langle g\right|+\frac{1}{2}\left(e^{-i\chi }+g^{2}e^{i\chi }C^{+}\right)\sin\theta |e\rangle \langle g|\\ \; & +\frac{1}{2}\left(e^{i\chi }+g^{2}e^{-i\chi }C^{-}\right)\sin\theta |g\rangle \langle e|+\left(\sin^{2}\frac{\theta }{2}+g^{2}TR_{\mathrm{acc}}^{+}\cos^{2}\frac{\theta }{2}\right)|e\rangle \langle e|], \end{array}$$

where

$$\begin{array}{cc} N & =1+g^{2}TR_{\mathrm{acc}}^{+}\cos^{2}\frac{\theta }{2}+g^{2}TR_{\mathrm{acc}}^{-}\sin^{2}\frac{\theta }{2},\\ C^{\pm } & =\int_{-\infty }^{\infty }d\tau \int_{-\infty }^{\infty }d\tau^{\prime }\Theta \left(\tau \right)\Theta \left(\tau^{\prime }\right)e^{\pm i\Omega (\tau +\tau^{\prime })}G^{+}\left(\tau -\tau^{\prime }\right). \end{array}$$

It should be noted that the expansion of the normalization factor ensures that the perturbative correction of the density matrix is traceless [20,22]. Hence, the accelerated qubit is expected to exhibit a loss of coherence in which the pure state $|\psi_{D}\rangle$ reduces to a statistical mixture after its interaction with the scalar field. It is important to note that the process of coherence loss requires a certain amount of time, known as the decoherence time $\tau_{d}$ [33]. According to the uncertainty relation ΔτΩ≳1, where Δτ represents the timescale for the transition between the two levels of the detector, it is evident that the interaction cannot take place within a time interval shorter than $\frac{1}{\Omega }$. This imposes a lower limit on the decoherence time, which must be taken into account in our theoretical approach to ensure the effective functioning of the detector. In the context of a two-level quantum system, the term “$l^{1}$ norm coherence” typically refers to the coherence between the two basis states of the system, which are commonly represented as $|g\rangle$ and $|e\rangle$. Coherence reflects the quantum property of superposition and interference between these states [34,35,36,37]. The $l^{1}$ norm coherence specifically quantifies coherence using the $l^{1}$ norm, also known as the Manhattan norm or the sum of absolute differences. Mathematically, for a two-level system, the $l^{1}$ norm coherence is given by the sum of absolute values of the off-diagonal elements of the density matrix ${\hat{\rho }}_{D}$ that describes the system:

(16) $$\begin{array}{c} Q_{l^{1}}\left({\hat{\rho }}_{D}\right)=\frac{\sin\theta }{N}\left[1+\frac{g^{2}Ta}{4\pi \sqrt{\pi }}e^{-\Omega^{2}T^{2}}\cos2\chi +O\left(g^{4}\right)\right], \end{array}$$

**Figure 2:**
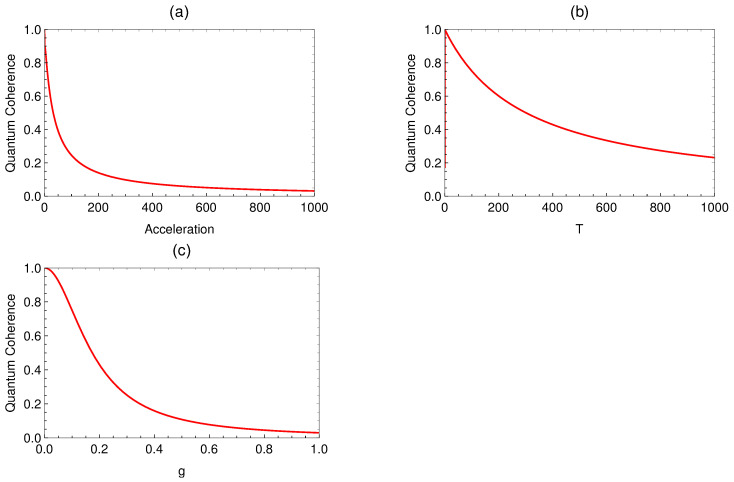
(**a**) $Q_{l^{1}}\left({\hat{\rho }}_{D}\right)$ as a function of the acceleration with g=0.1, T=100, θ=π/2, and χ=0. (**b**) $Q_{l^{1}}\left({\hat{\rho }}_{D}\right)$ as a function of the interaction time *T* with g=0.1, a=10, θ=π/2, and χ=0. (**c**) $Q_{l^{1}}\left({\hat{\rho }}_{D}\right)$ as a function of the coupling constant *g* with T=100, a=10, θ=π/2, and χ=0. The parameter Ω is fixed as Ω=1.

The $l^{1}$ norm coherence quantifies the magnitude of the off-diagonal elements of the density matrix, which correspond to the probability amplitudes of transitioning between the two basis states. A higher l1-norm coherence indicates a greater degree of superposition and quantum interference. In Figure 2a below, we have plotted (16) as a function of acceleration. We observe that when the acceleration is sufficiently low, the quantum coherence maintains a value that is close to its maximum, i.e., $Q_{l^{1}}\left({\hat{\rho }}_{D}\right)=1$. This is due to the fact that at very low acceleration, the temperature of the Unruh thermal bath is low. Consequently, only a few particles possess sufficient energy to interact with the qubit. On the other hand, when the accelerations grow arbitrarily, the qubit experiences high Unruh temperatures, and its quantum coherence decreases monotonically.

Figure 2b depicts the quantum coherence as a function of the interaction time and reveals an asymptotic decoherence pattern with almost sub-exponential decay. It can be observed that when *T* decreases, the quantum coherence grows, indicating higher loss of coherence for a longer interaction time regime. This asymptotic regime occurs when $T\gg \frac{1}{\Omega }$: meaning that the interaction time is much longer compared to the timescale for transitions in the detector.

Figure 2c illustrates the behavior of quantum coherence as a function of the coupling constant *g*. It is evident from the plot that the quantum coherence decreases monotonically as the parameter *g* increases. This indicates that the loss of coherence, induced by Unruh thermal noise, is amplified in the strong coupling regime. The dynamics of the detection probability in the excited and ground states, as well as the asymptotic convergence of the density operator to mixed states, were investigated in references [38,39]. Additionally, in superconducting devices [40,41], the same final state can be generated exponentially due to various factors, including interactions with a thermal bath. In practical terms, the $l^{1}$ norm coherence plays a significant role in characterizing the behavior of quantum systems, especially in applications such as quantum information processing and quantum metrology. It serves as a useful measure of the coherence present in a quantum state and can be utilized as a resource for performing various quantum operations, including quantum detection.

## 4. Setup B: Quantum Interferometric Circuit

One way to indirectly measure a system’s properties involves implementing a quantum scattering circuit that utilizes quantum interferometry [42] to extract information on a specific component of the system through the examination of the physical attributes of a probe. Various applications of quantum scattering circuits include testing the Legget–Garg inequality [43], measuring correlation functions in Fano–Anderson problem simulations [44], determining discrete Wigner functions [45,46], experimentally reconstructing work distribution [47], etc. A quantum scattering circuit involves a controlled interaction between a single qubit, referred to as a probe, and the system of interest.

**Figure 3:**
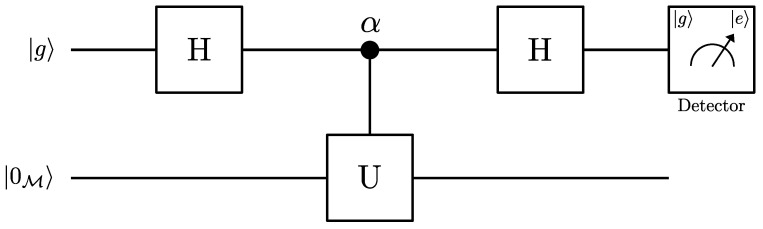
Schematic diagram of a quantum interferometric circuit in Unruh effect scenario.

In order to get a better understanding of the connection between the Unruh effect and quantum coherence, let us introduce the quantum network represented by the quantum scattering circuit of Figure 3. The quantum scattering circuit consist of the following steps: First, a single qubit (called the probe) is prepared in a known initial state. Then, the first Hadamard gate puts the probe in the superposition state. Next, a unitary operator *U* and a phase shift are simultaneously applied to the system. In this step, the probe qubit interacts with a system (e.g., a massless scalar field) in a controlled way. Finally, the qubit passes through the second Hadamard gate. The coupling between the uniformly accelerated detector and the scalar field, initially in the vacuum state, modifies the interference pattern. In this context, the scattering circuit can be used to estimate the Unruh temperature and the coherence loss induced by the Unruh thermal bath.

Let us suppose that initially the system (probe qubit plus field) is prepared in the state $|\psi_{\mathrm{in}}\rangle =|0_{M}\rangle ⊗|g\rangle$, where $|0_{M}\rangle$ is the Minkowski vacuum state of the field defined in the inertial laboratory frame. The action of the first Hadamard gate transforms the |g〉 state to the superposition state:

(17) $$\begin{array}{c} |\psi_{1}\rangle \to \frac{1}{\sqrt{2}}|0_{M}\rangle ⊗[|g\rangle +|e\rangle ]. \end{array}$$

Then, the probe qubit is linearly accelerated and is coupled to a massless scalar field. The coupling between the uniformly accelerated qubit and the scalar field is governed by the $\hat{U}$ operator induced by the Hamiltonian (1). As a result, the interaction between the uniformly accelerated qubit and the massless scalar field produces the transformations (13). For our purposes, we are interested in observing only the effects of the acceleration. For this, we consider that the transition probability from the excited to the ground state is negligible when the non-accelerated qubit interacts with the scalar field in the vacuum state. This is consistent with an atom–field interaction in the weak coupling regime and for very short interaction times [32]. In addition, the probability amplitude of finding the qubit in |g〉 or |e〉 between the Hadamard gates accumulates a quantum phase. Thus, a phase shift α is introduced by the phase-shift gate if the qubit is in |e〉. Therefore, the simultaneous application of the unitary operator $\hat{U}$ and the phase shift in Equation (17) transforms the state of the system to

$$\begin{array}{cc} |\psi_{2}\rangle & \to \frac{1}{\sqrt{2}}\left[|0_{M}\rangle ⊗(|g\rangle +e^{i\alpha }\left|e\rangle )-ig\Phi \left(\tau \right)\right|0_{M}\rangle ⊗|e\rangle -ige^{i\alpha }\Phi^{*}\left(\tau \right)|0_{M}\rangle ⊗|g\rangle \right]. \end{array}$$

Finally, after the operation of the second Hadamard gate, the final state of the system reads

$$\begin{array}{cc} |\psi_{3}\rangle & \to e^{i\frac{\alpha }{2}}\left\{\right|0_{M}\rangle ⊗\left[\cos\frac{\alpha }{2}|g\rangle -i\sin\frac{\alpha }{2}|e\rangle \right]-\frac{ige^{-i\frac{\alpha }{2}}}{2}\Phi \left(\tau \right)|0_{M}\rangle ⊗[|g\rangle \\ \; & -\left|e\rangle \right]-\frac{ige^{i\frac{\alpha }{2}}}{2}\Phi^{*}\left(\tau \right)|0_{M}\rangle ⊗[|g\rangle +|e\rangle ]\}. \end{array}$$

Tracing out the field’s degrees of freedom, we find the reduced density matrix of the qubit:

ρ^A=Tr|0M〉[|ψ3〉〈ψ3|],=N¯−1{cos2α2+g24TRacc−+TRacc++2Re(eiαC+)|g〉〈g|+2isinα+g24TRacc−−TRacc++2iIm(eiαC+)|e〉〈g|+−2isinα+g24TRacc−−TRacc+−2iIm(eiαC+)|g〉〈e|+sin2α2+g24TRacc−+TRacc+−2Re(eiαC+)|e〉〈e|},

where $\bar{N}=1+\frac{g^{2}}{2}TR_{\mathrm{acc}}^{-}+\frac{g^{2}}{2}TR_{\mathrm{acc}}^{+}.$

### 4.1. Quantum Coherence and Visibility

Let us explore the properties of the reduced density matrix of the qubit. From ${\hat{\rho }}_{A}$, we find that the probability of measuring the detector in the |g〉 state is given by

Pg=cos2α2+g24TRacc−+TRacc++2Re(eiαC+)1+g2ΩT4πcothπΩa+g2aTe2πΩae2πΩa−131−e2πΩa+πΩae2πΩa+1.

When T≫1/Ω, we obtain

$$\begin{array}{c} P_{g}\approx \frac{\cos^{2}\frac{\alpha }{2}+\frac{g^{2}\Omega T}{8\pi }\coth\left(\frac{\pi \Omega }{a}\right)}{1+\frac{g^{2}\Omega T}{4\pi }\coth\left(\frac{\pi \Omega }{a}\right)}. \end{array}$$

The fringe visibility for the interference pattern is defined by

$$\begin{array}{c} V=\frac{P_{g}^{\max}-P_{g}^{\min}}{P_{g}^{\max}+P_{g}^{\min}}, \end{array}$$

where the max/min values are calculated with respect to phase α. The maximum and minimum values of the probability $P_{g}$ occur when α=0 and α=π, respectively. Calculation demonstrates that the visibility is determined as

(18) $$\begin{array}{c} V=\frac{1+\frac{g^{2}Ta}{4\pi \sqrt{\pi }}e^{-\Omega^{2}T^{2}}}{1+\frac{g^{2}\Omega T}{4\pi }\coth\left(\frac{\pi \Omega }{a}\right)+\frac{g^{2}}{aT}\frac{e^{\frac{2\pi \Omega }{a}}}{{\left(e^{\frac{2\pi \Omega }{a}}-1\right)}^{3}}\left[1-e^{\frac{2\pi \Omega }{a}}+\frac{\pi \Omega }{a}\left(e^{\frac{2\pi \Omega }{a}}+1\right)\right]}. \end{array}$$

Note that for T≫1/Ω, the second term in the numerator and the last term in the denominator go to zero, and we get

(19) $$\begin{array}{c} V\approx \frac{1}{1+\frac{g^{2}\Omega T}{4\pi }\coth\left(\frac{\pi \Omega }{a}\right)}. \end{array}$$

These results provide support for the hypothesis that the Unruh effect destroys the quality of the interference pattern. The $l^{1}$ norm of the coherence of ${\hat{\rho }}_{A}$ is given by

(20) $$\begin{array}{c} Q_{l^{1}}\left({\hat{\rho }}_{A}\right)=\frac{\sin\alpha \left[1+\frac{g^{2}Ta}{4\pi \sqrt{\pi }}e^{-\Omega^{2}T^{2}}+O\left(g^{4}\right)\right]}{1+\frac{g^{2}\Omega T}{4\pi }\coth\left(\frac{\pi \Omega }{a}\right)+\frac{g^{2}}{aT}\frac{e^{\frac{2\pi \Omega }{a}}}{{\left(e^{\frac{2\pi \Omega }{a}}-1\right)}^{3}}\left[1-e^{\frac{2\pi \Omega }{a}}+\frac{\pi \Omega }{a}\left(e^{\frac{2\pi \Omega }{a}}+1\right)\right]}. \end{array}$$

By comparing (18) and (20), we obtain the following relation: $Q_{l^{1}}\left({\hat{\rho }}_{A}\right)\approx \sin\alpha V$. Like visibility, quantum coherence captures the wave nature of the probe qubit. Note that while coherence is an intrinsic feature of an isolated quantum state, Unruh thermal noise on a uniformly accelerated qubit can impact it due to the interaction with the scalar field.

### 4.2. Which-Path Distinguishability

**Figure 4:**
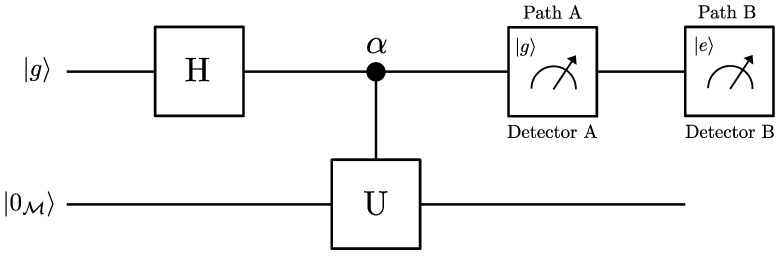
Schematic diagram of path information in Unruh effect scenario.

In order to obtain which-way information, we remove the second Hadamard gate from the interferometer setup illustrated in Figure 3 and include two detectors. According to Figure 4, the qubit, initially prepared in its ground state |g〉, is transformed to a superposition state by the first Hadamard. It is well known that the Hadamard gate is the quantum network equivalent of a beamspliter in a Mach-Zehnder interferometer. Two path detectors are inserted just after the linear acceleration zone to probe the which-path information of the qubit. When the internal state of the qubit is found to be |g〉 in detector A, the qubit is transmitted along path A; otherwise, it is reflected along path B. Thus, the which-way information can be stored in the internal states of the qubit.

The path distinguishability characterizes the particle nature of the qubit and its quantifier via the difference between the probabilities of detecting the qubit in detector A (path A) or in detector B (path B):

(21) $$\begin{array}{c} D=\frac{|w_{A}-w_{B}|}{w_{A}+w_{B}}, \end{array}$$

where the probability of finding the field in the state |F〉 and the qubit in the |g〉 state (path A) is given by

$$\begin{array}{cc} w_{A} & =\sum_{F}{\left|\langle F,g|0_{M},g\rangle \right|}^{2}=\frac{1}{2}\left[1+g^{2}TR_{\mathrm{acc}}^{-}\right]. \end{array}$$

Similarly, the probability of finding the field in the state |F〉 and the qubit in the |e〉 state (path B) is

$$\begin{array}{cc} w_{B} & =\sum_{F}{\left|\langle F,e|0_{M},e\rangle \right|}^{2}=\frac{1}{2}\left[1+g^{2}TR_{\mathrm{acc}}^{+}\right]. \end{array}$$

Thus, the path distinguishability becomes

(22) $$\begin{array}{cc} D & =\frac{g^{2}T}{2\bar{N}}\left|R_{\mathrm{acc}}^{-}-R_{\mathrm{acc}}^{+}\right|,\\ \; & =\frac{\frac{g^{2}\Omega T}{4\pi }}{1+\frac{g^{2}\Omega T}{4\pi }\coth\left(\frac{\pi \Omega }{a}\right)+\frac{g^{2}}{aT}\frac{e^{\frac{2\pi \Omega }{a}}}{{\left(e^{\frac{2\pi \Omega }{a}}-1\right)}^{3}}\left[1-e^{\frac{2\pi \Omega }{a}}+\frac{\pi \Omega }{a}\left(e^{\frac{2\pi \Omega }{a}}+1\right)\right]}. \end{array}$$

When T≫1/Ω, we obtain

(23) $$\begin{array}{c} D=\frac{\frac{g^{2}\Omega T}{4\pi }}{1+\frac{g^{2}\Omega T}{4\pi }\coth\left(\frac{\pi \Omega }{a}\right)}. \end{array}$$

This result shows that the Unruh effect has an impact on the particle nature of the qubit when it is uniformly accelerated.

### 4.3. Complementarity Relation

We are now ready to investigate the effect of the Unruh thermal bath on the wave–particle duality relation. By including the Unruh effect in the interferometric setup, we have the expressions for visibility and path distinguishability in Equations (18) and (22), respectively. By squaring and adding them, we obtain the complementarity relation as

(24) $$\begin{array}{c} C=V^{2}+D^{2}\leq 1. \end{array}$$

Note that the equal sign holds when both the qubit and the quantum field are in a pure state. Equation (24) shows a mutually exclusive relation between wave-like and particle-like behavior. In other word, it is the duality relation for a Ramsey interferometer. For T≫1/Ω, we get

(25) $$\begin{array}{c} C\approx \frac{1}{1+\frac{g^{2}\Omega T}{4\pi }\coth\left(\frac{\pi \Omega }{a}\right)}+O\left(g^{4}\right). \end{array}$$

This result implies that the Unruh effect inevitably disturbs the internal state of the accelerated qubit and causes some loss of wave–particle duality information.

## 5. Numerical Analysis

In this section, we perform a joint numerical analysis of visibility, path distinguishability, and complementarity for comparison. Our aim is to explore the behavior of wave–particle duality under the Unruh effect.

**Figure 5:**
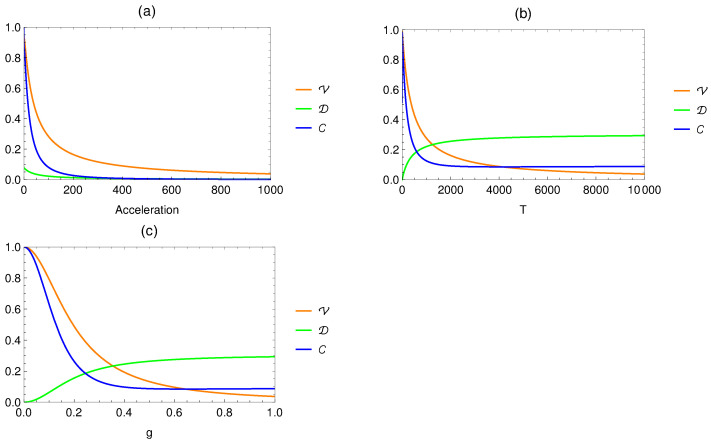
(**a**) Plots of visibility, path distinguishability, and complementarity as a function of the acceleration with g=0.1 and T=100. (**b**) Plots of visibility, path distinguishability, and complementarity as a function of the interaction time *T* with g=0.1 and a=10. (**c**) Plots of visibility, path distinguishability, and complementarity as a function of the coupling constant *g* with T=100 and a=10. The parameter Ω is fixed as Ω=1.

In Figure 5a, we plot the behavior of visibility, path distinguishability, and complementarity as a function of the acceleration for fixed parameters g=0.1 and T=100. It is shown that both visibility and path distinguishability—and hence, the complementarity relation—decrease with increasing acceleration. This suggests that in the regime of arbitrary accelerations, the qubit experiences high Unruh temperatures and the Unruh thermal bath has a significant impact on the its wave–particle properties. In this case, both visibility and path distinguishability vanish and no information about the wave and particle aspects of the qubit can be obtained. As noticed by Jakob and Bergou [48], this lack of knowledge about the probe system is due to another intriguing quantum feature: namely, quantum entanglement. This suggests that the information is being shared with the massless scalar field.

In Figure 5b, we plot the visibility, path distinguishability, and complementarity as a function of the interaction time *T*. We can see that the visibility and complementarity decrease as the interaction time grows. In contrast, the path distinguishability approaches its maximum value if the time interaction is long enough. Similarly, Figure 5c shows that visibility, path distinguishability, and complementarity vary by changing the values of the coupling constant *g*. These results mean that we have partial particle behavior for the probe qubit inside the interferometer when there is a longer interaction time or in a strong coupling regime.

## 6. Conclusions

It is well known that one of the most non-classical manifestations of quantum mechanics is wave–particle duality or interferometric complementarity. In this paper, we investigate the effect of an Unruh thermal bath on the wave–particle properties of a uniformly accelerated qubit. Our analysis suggests that the creation of particles by the acceleration serves as the “environment” of the qubit, leading to a loss of information about the wave and particle aspects of the qubit inside the interferometer. We show that the Unruh effect can cause noticeable loss of coherence, resulting in reduced fringe visibility, path distinguishability, and complementarity. This is so because the interaction between the qubit and the scalar field causes them to correlate, leading to an irreversible transfer of information from the qubit to the Unruh thermal bath. In particular, when a→∞, the complementarity C→0, indicating a maximally mixed state of the probe qubit. For very high acceleration, the qubit and scalar field become maximally entanglement (see discussion in [48]). In addition, we notice that strong coupling and a longer interaction time have significant effects on the wave–particle properties of the probe qubit. Our investigations help to provide understanding of the consequences of decoherence induced by the Unruh effect in a hypothetical experimental test of the wave–particle duality of a uniformly accelerated qubit. This also opens the possibility to investigate other scenarios, such as Hawking radiation, the dynamical Casimir effect, and non-uniformly accelerated qubits, that may affect the wave–particle duality.

## Data Availability

The data can be obtained via correspondence upon reasonable request.
